# The assessment of femoral shaft morphology in the sagittal plane in Chinese patients with osteoarthritis—a radiographic analysis

**DOI:** 10.1186/s13018-017-0626-8

**Published:** 2017-08-30

**Authors:** Zhengyuan Bao, Liang Qiao, Jianghui Qin, Jiacheng Xu, Sheng Zhou, Dongyang Chen, Dongquan Shi, Jin Dai, Yao Yao, Qing Jiang, Zhihong Xu

**Affiliations:** 1 Department of Sports Medicine and Adult Reconstructive Surgery, Drum Tower Hospital, School of Medicine, Nanjing University, 321 Zhongshan Road, Nanjing, Jiangsu 210008 China; 2 Laboratory for Bone and Joint Disease, Model Animal Research Center (MARC), Nanjing University, Nanjing, Jiangsu 210093 China; 3 The Affiliated Hospital of Nantong University, Nantong, Jiangsu 226000 China

**Keywords:** Femoral shaft bowing, Sagittal plane, Radiographic analysis, Knee osteoarthritis, Total knee arthroplasty

## Abstract

**Background:**

The purpose of this study was to analyze femoral shaft sagittal parameters in Chinese osteoarthritis (OA) patients undergoing total knee arthroplasty (TKA) and identify whether the parameters in the coronal plane could be predictors of those in the sagittal plane.

**Methods:**

Standard long-standing anteroposterior and femoral lateral radiographs of 50 lower limbs in 50 Chinese OA patients were included. Sagittal femoral bowing angle (sFBA), angle between femoral distal anterior cortex axis and sagittal mechanical axis (DACSMA), angle between femoral distal anterior cortex axis and sagittal distal anatomic axis (DACSDAA), and angle between femoral sagittal mechanical axis and sagittal distal anatomic axis (SMADAA) were measured. Then the relationship between femoral shaft parameters in the sagittal and coronal planes were identified, including coronal femoral bowing angle (cFBA), valgus angle, hip-knee-ankle angle (HKA), length of femur (LF), femoral offset, femoral neck stem angle (FNS), and mechanical lateral distal femoral angle (mLDFA). A two-sided Pearson correlation coefficient was obtained to identify the correlations between parameters in the coronal and sagittal planes. *P* values <0.05 were considered statistically significant.

**Results:**

The mean sFBA was 15.08° ± 3.79°, the mean DACSMA was 1.35° ± 2.70°, the mean DACSDAA was −2.66° ± 2.05°, and the mean SMADAA was 4.01° ± 2.55°. No correlation between parameters in the coronal and sagittal planes was found.

**Conclusions:**

In this study, the discreteness of DACSMA, DACSDAA, and SMADAA in Chinese OA patients is high and this may affect the position of femoral prosthesis after TKA using the conventional intramedullary device. No parameters in the coronal plane are found correlated with those in the sagittal plane.

**Trial registration:**

Researchregistry2337

## Background

As a major source of lower limb pain and disability, knee osteoarthritis (OA) generates great impacts on patients’ quality of life and brings a heavy burden for public health system [1]. For severe knee OA, total knee arthroplasty (TKA) is the preferred treatment. Postoperative implant alignment is an important factor related to the outcomes of TKA [2]. However, the present conventional intramedullary device shows lots of drawbacks in practice. It may cause the coronal malalignment due to the differences of femoral shaft shape. What is more, it ignores the importance of good alignment in the sagittal plane. So the assessing of femur shaft morphology in both coronal and sagittal planes is important preoperatively.

The effect of femoral shaft bowing (FSB) on the position of implant in TKA has drawn more and more attention especially from Asian OA patients. However, the definition for FSB has not been well established. Akamatsu defined coronal femoral bowing angle (cFBA) >5° in the coronal plane as coronal femoral shaft bowing (cFSB) and sagittal femoral bowing angle (sFBA) of >11° in the sagittal plane was defined as sagittal femoral shaft bowing (sFSB) [3]. Previous study also found FSB had racial specificity and Asians were more susceptible [4]. It is related to higher prevalence and faster progression of knee OA [5]. Furthermore, severe FSB may affect the implant position during TKA surgery. The conventional intramedullary femoral cut system sets femoral coronal mechanical axis (cMA) by referring the intramedullary rod and the valgus angle between cMA and coronal anatomical axis of the femur. The best outcome of coronal alignment is limited within 3° of cMA [5]. cFBA has been reported to be associated with valgus angle positively, and if cFBA increases, valgus angle will be larger [6]. So cFBA is also related to postoperative limb and implant alignment [7].

Unlike the recognized results in the coronal plane, there is no unified peri-operative alignment assessment system of femur in the sagittal plane. It has been shown sFBA is associated with the degree of femoral component flexion [8]. An overly flexional position will limit knee extension and result in posterior insert wear caused by impingement between the polyethylene insert and the intercondylar box in TKA using post-cam mechanism [9]. And an over-extensional position may contribute to a postoperative supracondylar femoral fracture [10]. So cFSB and sFSB are both of important clinical meaning.

Considering the negligent assessment of the femoral morphology in the sagittal plane before TKA in China, the purpose of this study was as follows: first, to analyze different parameters of femoral shaft in the sagittal plane of Chinese people with knee OA undergoing TKA; second, to identify which parameters in the coronal plane could be predictors of those in the sagittal plane using radiographs.

## Methods

### Patients

Chinese patients with knee OA who underwent TKA from May, 2015, to July, 2016, in our surgical team (Xu) were reviewed. The preoperative standard long-standing anteroposterior and femoral lateral radiographs [11] were examined in all the patients. When taking long-standing anteroposterior radiographs, two lower limbs were rotated neutrally with the patellae facing forwards and the beam tube centered at the knee [6]. The key point of taking femoral lateral radiographs was to make sure the beam tube was tilted 15° to aim the midpoint of the patients’ thigh directly [11]. We excluded limbs which had a prior fracture and prior knee or hip arthroplasty, also those with nonstandard films. Totally, 50 patients were enrolled.

### Radiographic assessment

**Fig. 1:**
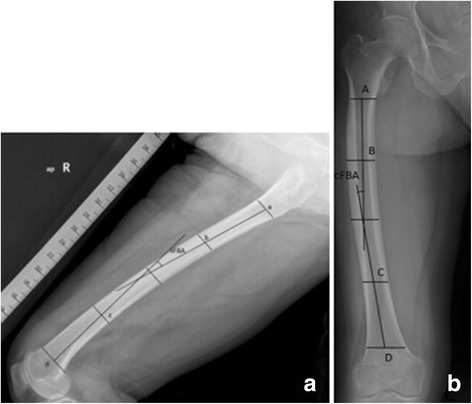
**a**, **b** The femoral shaft was divided into four equal parts in both coronal and sagittal planes. The proximal end was the lower border of the lesser trochanter and the distal end was the junction between the shaft and the condylar region. sFBA and cFBA were angles between midlines drawn in the proximal and distal quarter segments of the femoral shaft. **a** Points *a*, *b*, *c*, *d* were the midpoints of medullary cavity in the sagittal plane. **b** Points *A*, *B*, *C*, *D* were the midpoints of medullary cavity in the coronal plane

**Fig. 2:**
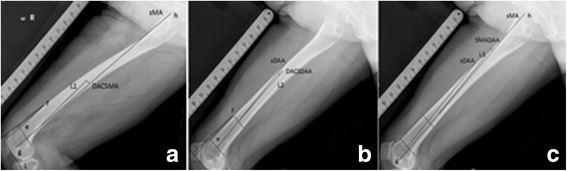
**a** The DACA was the line connecting two points on the anterior cortex at 5 cm (point e) and 10 cm (point f) proximal to L (L was the tangent line of distal femur parallel to the knee line, the knee line was the junction between the shaft and the condylar region). sMA was the line connecting DPIN (point g) and the center of femoral head (point h). DACSMA was the angle between sMA and DACA, and L1 was parallel to DACA. **b** sDAA was the midline drawn in the distal quarter of femoral shaft. DACSDAA was the angle between DACA and sDAA, and L2 was parallel to DACA. **c** SMADAA was the angle between sMA and sDAA, and L3 was parallel to sDAA

**Fig. 3:**
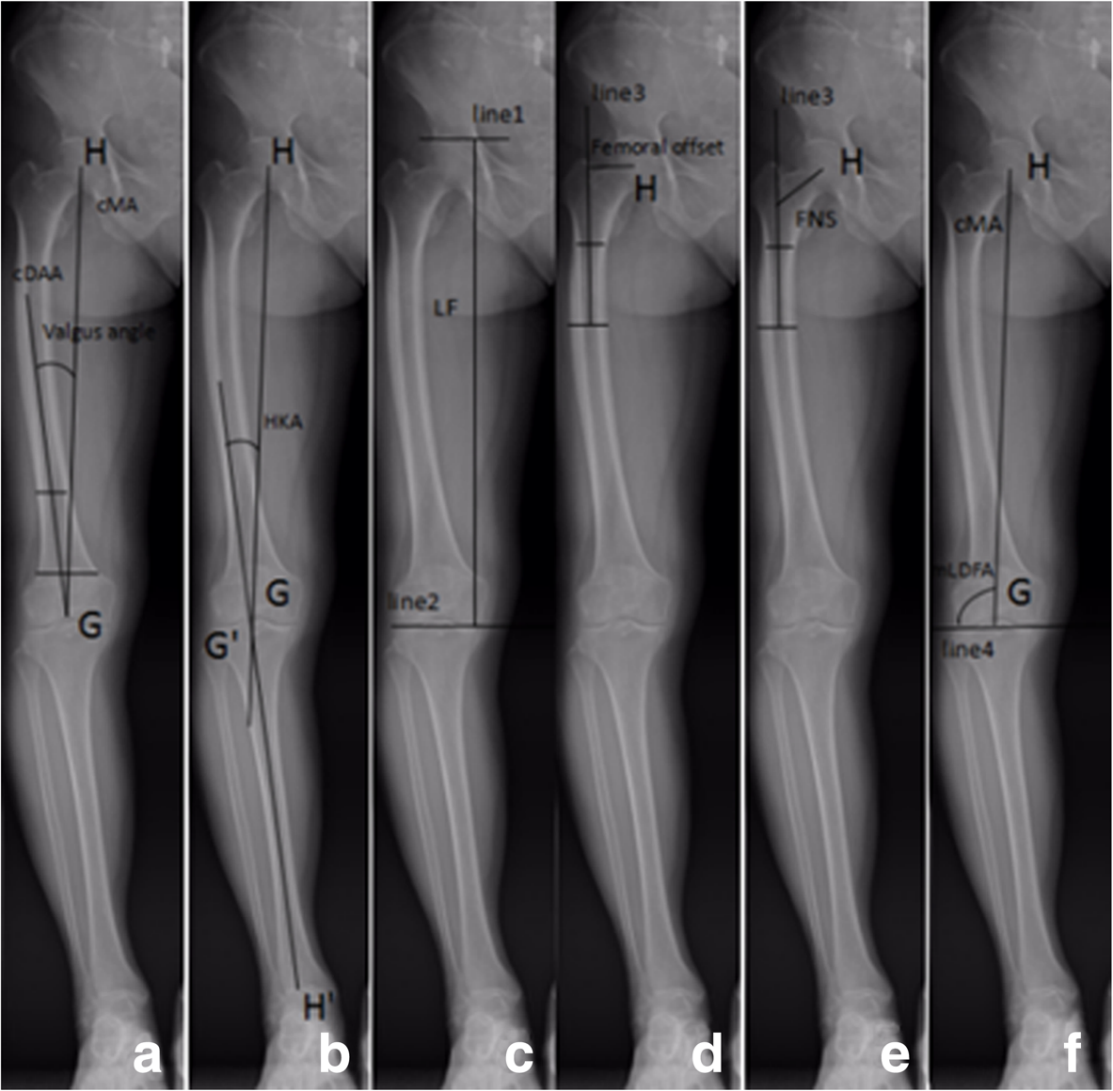
**a** The valgus angle was the angulation between femoral cMA (point H: the center of femoral head; point G: DPIN) and cDAA (the line connecting DPIN to upper midpoint of the distal quarter segment of the shaft). **b** HKA was the angle between femoral cMA and tibial cMA (point G’: the midpoint of the medial and lateral tibial eminences; point H′: the midpoint of the talus dome). **c** LF was the distance between two horizontal lines (line1 and line2) covering the entire femur. **d** The femoral offset was the vertical distance from point H to the midline drawn in the proximal quarter of femoral shaft (line3). **e** FNS was the angle between the two midlines drawn in the proximal quarter of femoral shaft and the femoral neck. **f** mLDFA was the lateral angle between femoral cMA and the knee line, the distal femur articular surface (line4)

All radiographic measurements were obtained from true long-standing anteroposterior and femoral lateral radiographs using Picture Archiving Communication System (PACS, FIRSTECH, Hefei, Anhui, China). We only examined the operated limb. In femoral lateral radiographs, the femoral shaft was divided into four equal parts in the sagittal plane [3]. The proximal end of the diaphysis was the lower border of the lesser trochanter and the distal end was the junction between the shaft and the condylar region. The angle between the midlines drawn in the proximal and distal quarter segments was defined as sFBA. Positive values meant femoral anterior bowing and negative values meant posterior bowing (Fig. 1a). There was no agreed definition of sagittal mechanical axis (sMA) [11–13]. Here we defined sMA as the line connecting the center of femoral head and the deepest point of the intercondylar notch (DPIN). In the femoral lateral radiograph, DPIN was the end of Blumensaat’s line [11]. DACSMA was defined as the abbreviation of the angle between femoral distal anterior cortex axis (DACA) [11] and sMA (Fig. 2a). A positive value meant DACA was in flexion to sMA and a negative value meant DACA was in extension. DACSDAA was defined as the angle between DACA and sagittal distal anatomic axis (sDAA) (Fig. 2b); sDAA was the midline drawn in the distal quarter of femoral shaft. If DACA was in flexion to sDAA, this value was positive, otherwise this value was negative. SMADAA was defined as the angle between sDAA and sMA (Fig. 2c). Positive values meant sDAA was in flexion to sMA, otherwise sDAA was in extension to sMA. In long-standing anteroposterior radiographs, similar to the partition method in the sagittal plane, the angle between the midlines drawn in the proximal and distal quarter segments of the femoral shaft was defined as cFBA. Positive values meant femoral lateral bowing and negative values meant medial bowing (Fig. 1b). The valgus angle was defined as the angulation between femoral cMA and coronal distal anatomic axis (cDAA) (Fig. 3a). Femoral cMA was a line connecting the center of femoral head to DPIN. Femoral cDAA was the midline drawn in the distal quarter of femoral shaft in the coronal plane. In the preoperative measurement of the valgus angle, femoral cMA and cDAA shared the same end point, the entry point of intramedullary rod. Different surgeons prefer different entry points and here we chose DPIN as the entry point [11]. Then we took the line connecting DPIN to upper midpoint of the distal quarter segment of the shaft as cDAA [6]. Hip-knee-ankle angle (HKA) was the angle between femoral cMA and tibial cMA (Fig. 3b). Tibial cMA was a line connecting the midpoint of the medial and lateral tibial eminences and the midpoint of the talus dome. If the knee was varus, this value was positive, otherwise HKA was negative. The length of femur (LF) was the distance between two horizontal lines covering the entire femur (Fig. 3c). The femoral offset was the vertical distance from the center of femoral head to the midline drawn in the proximal quarter of femoral shaft [14] (Fig. 3d). The femoral neck stem angle (FNS) was the angle between the two midlines drawn in the proximal quarter of femoral shaft and the femoral neck [14](Fig. 3e). mLDFA was the lateral angle between femoral cMA and the knee line, the distal femur articular surface [13](Fig. 3f). All parameters and corresponding definitions are summarized in Table 1.

**Table 1:** Radiographic parameters and corresponding definitions

Radiographic parameters	Definition
sFBA	The angle between the midlines drawn in the proximal and distal quarter segments in the sagittal plane
DACSMA	The angle between femoral distal anterior cortex axis and sagittal mechanical axis
DACSDAA	The angle between distal anterior cortex axis and sagittal distal anatomic axis
SMADAA	The angle between sagittal distal anatomic axis and sagittal mechanical axis
cFBA	The angle between the midlines drawn in the proximal and distal quarter segments of the femoral shaft in the coronal plane
Valgus angle	The angle between femoral coronal mechanical axis and coronal distal anatomic axis
HKA	The angle between femoral coronal mechanical axis and tibial coronal mechanical axis
LF	The distance between two horizontal lines covering the entire femur
Femoral offset	The vertical distance from the center of femoral head to the midline drawn in the proximal quarter of femoral shaft
FNS	The angle between the two midlines drawn in the proximal quarter of femoral shaft and the femoral neck
mLDFA	The lateral angle between femoral coronal mechanical axis and the knee line, the distal femur articular surface

### Statistical analysis

Data were analyzed using SPSS 19.0 (SPSS Inc., Chicago, IL, USA). All measurements were taken by two researchers (Bao & Qiao) with no communication with each other. The two surgeons performed the measurements twice in 2 weeks. The degree of measurement reliability was assessed by intraclass correlation coefficients (ICC). The 95% confidence intervals of ICC for intraobserver and interobserver reliability were both >0.85. As the reproducibility of all measurements was high, the mean values of measurements made by one of the researchers (Bao) were used for all subsequent analyses. All the data were expressed as the mean ± standard deviation (SD) and range. A two-sided Pearson correlation coefficient was obtained to identify the correlations between parameters in the coronal and sagittal planes. *P* values <0.05 were considered statistically significant.

## Results

The mean sFBA, DACSMA, DACSDAA, and SMADAA were 15.08° ± 3.79° (range 7.28°–25.02°), 1.35° ± 2.70° (range −5.55°–7.21°), −2.66° ± 2.05° (range −8.63°–1.45°), 4.01° ± 2.55° (−2.23°–10.54°), respectively. The mean cFBA, valgus angle, HKA, LF, femoral offset, FNS, and mLDFA were 4.87° ± 5.23° (−4.45°–18.53°), 5.87° ± 2.50° (1.54°–12.40°), 5.65° ± 4.96° (−7.64°–14.17°), 41.85 cm ± 2.55 cm (37.08 cm–50.06 cm), 3.89 cm ± 0.50 cm (2.98 cm–5.75 cm), 124.31° ± 7.43° (105.42°–141.25°), 88.22° ± 2.99° (78.96°–94.47°), respectively (Table 2). DACSMA and SMADAA correlated positively with sFBA (*r* = 0.563, *p* = 0.001; *r* = 0.840, *p* = 0.001; respectively). DACSDAA correlated negatively with sFBA (*r* = −0.301, *p* = 0.033) (Table 3). The correlation between parameters in the coronal and sagittal planes was poor (data not shown).

**Table 2:** Summary of the measured parameters

	*N*	Minimum	Maximum	Mean	SD
Age (year)	50	42.00	83.00	69.50	8.42
Weight (kg)	50	45.00	87.00	67.96	10.26
Height (m)	50	1.49	1.80	1.61	0.07
BMI (kg/m^2^)	50	18.37	35.11	26.39	4.00
cFBA (°)	50	−4.45	18.53	4.87	5.23
Valgus angle (°)	50	1.54	12.40	5.87	2.50
HKA (°)	50	−7.64	14.17	5.65	4.96
LF (cm)	50	37.08	50.06	41.85	2.55
Femoral offset (cm)	50	2.98	5.75	3.89	0.50
FNS (°)	50	105.42	141.25	124.31	7.43
mLDFA (°)	50	78.96	94.47	88.22	2.99
sFBA (°)	50	7.28	25.02	15.08	3.79
DACSMA (°)	50	−5.55	7.21	1.35	2.70
DACSDAA (°)	50	−8.63	1.45	−2.66	2.05
SMADAA (°)	50	−2.23	10.54	4.01	2.55

**Table 3:** Pearson correlation coefficients between sFBA, DACSMA, DACSDAA, and SMADAA

		sFBA	DACSMA	DACSDAA	SMADAA
sFBA	Pearson correlation coefficient	1	.563**	−.301*	.840**
	Significance (two-sided)		0	0.033	0
DACSMA	Pearson correlation coefficient	.563**	1	.453**	.695**
	Significance (two-sided)	0		0.001	0
DACSDAA	Pearson correlation coefficient	−.301*	.453**	1	−.326*
	Significance (two-sided)	0.033	0.001		0.021
SMADAA	Pearson correlation coefficient	.840**	.695**	−.326*	1
	Significance (two-sided)	0	0	0.021	

*p < 0.05

**p < 0.01

## Discussion

Considering most surgeons in China have paid more attention to the coronal shape of femur before TKA but ignored that in the sagittal plane. In this study, we tried to find predictors of sagittal parameters in the coronal plane. However, the correlation between parameters in the coronal and sagittal planes was poor. Moreover, age, height, weight, and body mass index (BMI) correlated with variants in the sagittal poorly too.

TKA is the mainstream treatment of severe OA and conventional intramedullary device is the most common femoral distal cut method during the operation. However, this device identifies femoral cMA indirectly during the operation and is restricted to the femoral shape. If the valgus angle or cFBA is too great, this method cannot ensure cMA and as a result, postoperative alignments will be in error and several clinical outcome scales will be inferior [7, 15, 16]. Moreover conventional intramedullary device cannot identify the femoral alignment in the sagittal plane. Recently, more and more surgeons have realized the significance of the femoral shape in the sagittal plane. Ko et al. thought sFBA was a risk factor for femoral implant flexion in conventional intramedullary TKA and notching in navigated TKA [8]. Nakahara et al. promoted an idea that sagittal femoral cutting error could change femoral anteroposterior sizing in TKA, for example, downsizing of the femoral component could occur if the distal osteotomy was performed in a flexed position [9]. And it is an agreement that an overly flexion position of femoral component will limit knee extension and result in polyethylene post wear caused by impingement between the polyethylene insert and the intercondylar box in TKA using post-cam mechanism [9]. Moreover, a hyperextension position may contribute to a postoperative supracondylar femoral fracture [10]. The alignments in the coronal and sagittal planes were equally important. Accordingly, our department senior surgeon Xu invented an extramedullary device and found this instrument could control both coronal and sagittal alignments better [17].

The present study found that in most limbs sDAA was in flexion to DACA (44 of 50 limbs) and this explained why the femoral implant was more likely in a flexed position by conventional intramedullary device [8]. Intramedullary method could not ensure sMA and femoral implant was more likely to be vertical to sDAA, as a result the alignment of prosthesis would be flexed to DACA. On the contrary, sMA was in extension to DACA in most limbs (33 of 50 limbs) and this explained why the femoral implant was more likely in an extended position using navigated method [8]. Navigated TKA could ensure sMA during the operation, and femoral implant was more likely to be vertical to sMA, then the alignment of prosthesis would be extended to DACA, resulting in anterior notching. Logically, sMA was more likely to extend to sDAA (45 of 50 limbs), and the mean angle was 4.01° ± 2.55°. This angle may explain the difference of femoral prosthesis position in the sagittal plane using conventional and navigated methods. We also found DACSMA and SMADAA were highly correlated with sFBA, so in patients with sFSB, it was easier to create anterior notching using a navigated method and the flexion difference of femoral prosthesis would be larger between conventional and navigated methods. Also it was notable that the discreteness of DACSMA, DACSDAA, and SMADAA was high. Most surgeons adjusted the femoral component flexion mainly referring to the DACA during TKA. But if DACSMA or DACSDAA was too great, the implant would be easily placed in a malposition. So we think that the assessing of femoral sagittal morphology routinely in patients undergoing TKA should be recommended.

In this study, we only took DAA as the track of intramedullary rod and did not identify the relationship between the real rod track and femoral axes. A previous study found that there were significant differences in the postoperative mechanical axes between the FSB (cFBA > 5°) and nonbowing groups after conventional TKA [18]. But there is still no recognized value classifying sFBA, so identifying this critical value is one of our focuses in the future. Further the present study only focused on preoperative radiographic features. Although there have been some researchers providing assessment methods of postoperative alignments, none of them combined alignments pre- and post-operatively together. So building a unified assessment system of lower limb alignments before and after the operation is also one of our future aims.

## Conclusions

In this study, the discreteness of DACSMA, DACSDAA, and SMADAA is high and no parameters in the coronal plane are correlated to those in the sagittal plane closely in Chinese OA patients. It is necessary to assess the morphological characteristics of femur in the sagittal plane before TKA.

## Abbreviations

BMI: Body mass index
cDAA: Coronal distal anatomic axis
cFBA: Coronal femoral bowing angle
cFSB: Coronal femoral shaft bowing
cMA: Coronal mechanical axis
DACA: Femoral distal anterior cortex axis
DACSDAA: Angle between femoral distal anterior cortex axis and sagittal distal anatomic axis
DACSMA: Angle between femoral distal anterior cortex axis and sagittal mechanical axis
DPIN: Deepest point of the intercondylar notch
FNS: Femoral neck stem angle
FSB: Femoral shaft bowing
HKA: Hip-knee-ankle angle
LF: Length of femur
mLDFA: The lateral angle between femoral coronal mechanical axis and the distal femur articular surface
OA: Osteoarthritis
sDAA: Sagittal distal anatomic axis
sFBA: Sagittal femoral bowing angle
sFSB: Sagittal femoral shaft bowing
sMA: Sagittal mechanical axis
SMADAA: Angle between femoral sagittal mechanical axis and sagittal distal anatomic axis
TKA: Total knee arthroplasty
